# Sequence Diversities of Serine-Aspartate Repeat Genes among *Staphylococcus aureus* Isolates from Different Hosts Presumably by Horizontal Gene Transfer

**DOI:** 10.1371/journal.pone.0020332

**Published:** 2011-05-20

**Authors:** Huping Xue, Hong Lu, Xin Zhao

**Affiliations:** 1 Department of Animal Science, McGill University, Montreal, Quebec, Canada; 2 State Key Laboratory of Genetic Engineering, School of Life Sciences, Fudan University, Shanghai, China; Baylor College of Medicine, United States of America

## Abstract

**Background:**

Horizontal gene transfer (HGT) is recognized as one of the major forces for bacterial genome evolution. Many clinically important bacteria may acquire virulence factors and antibiotic resistance through HGT. The comparative genomic analysis has become an important tool for identifying HGT in emerging pathogens. In this study, the Serine-Aspartate Repeat (Sdr) family has been compared among different sources of *Staphylococcus aureus* (*S. aureus*) to discover sequence diversities within their genomes.

**Methodology/Principal Findings:**

Four *sdr* genes were analyzed for 21 different *S. aureus* strains and 218 mastitis-associated *S. aureus* isolates from Canada. Comparative genomic analyses revealed that *S. aureus* strains from bovine mastitis (RF122 and mastitis isolates in this study), ovine mastitis (ED133), pig (ST398), chicken (ED98), and human methicillin-resistant *S. aureus* (MRSA) (TCH130, MRSA252, Mu3, Mu50, N315, 04-02981, JH1 and JH9) were highly associated with one another, presumably due to HGT. In addition, several types of insertion and deletion were found in *sdr* genes of many isolates. A new insertion sequence was found in mastitis isolates, which was presumably responsible for the HGT of *sdrC* gene among different strains. Moreover, the *sdr* genes could be used to type *S. aureus.* Regional difference of *sdr* genes distribution was also indicated among the tested *S. aureus* isolates. Finally, certain associations were found between *sdr* genes and subclinical or clinical mastitis isolates.

**Conclusions:**

Certain *sdr* gene sequences were shared in *S. aureus* strains and isolates from different species presumably due to HGT. Our results also suggest that the distributional assay of virulence factors should detect the full sequences or full functional regions of these factors. The traditional assay using short conserved regions may not be accurate or credible. These findings have important implications with regard to animal husbandry practices that may inadvertently enhance the contact of human and animal bacterial pathogens.

## Introduction

*Staphylococcus aureus (S. aureus)* is a highly adaptive and versatile gram-positive bacterium that presents growing and formidable global challenges for human and animal health concerns [1]. *S. aureus* can cause diseases ranging from superficial skin infections to life-threatening diseases such as pneumonia meningitis osteomyelitis endocarditis toxic shock syndrome (TSS) chest pain bacteremia and sepsis in human [2]. *S. aureus* also colonizes a range of other mammals including companion animals such as dogs, cats and horses [3], and livestock such as cows, pigs and goats [4], [5]. It can also colonize birds such as chickens and turkeys [6], [7], [8]. Thus, understanding the pathogenesis of *S. aureus* in different hosts is very important.

Comparative analyses of different *S. aureus* genomes have revealed that many strains have independently acquired genes from members of their surrounding microflora that confer antibiotic resistance and/or encode virulence factors [9], [10], [11]. The horizontal gene transfer (HGT) of mobile genetic elements (MGEs) among bacteria is the primary mode for the spread of antibiotic resistance and virulence factors in clinically important pathogens [11], [12]. MGEs consist of viruses, plasmids and associated elements (insertion sequences, transposons and integrons) that are either self-transmissible or use mobile plasmids and viruses as vehicles for their dissemination [13], [14]. A hypothesis that specific combinations of virulence factors encoded within MGEs are exchanged among strains by a “plug and play” mechanism has been proposed to explain occurrence of clones that are particularly well-adapted for causing certain diseases or infecting specific hosts [15], [16].

In comparison with the sequenced *S. aureus* strains associated with human infection, allelic variation in bovine strain RF122, also known as strain ET3-1, was high among virulence and surface-associated genes involved in host colonization, toxin production, iron metabolism, antibiotic resistance and gene regulation [16]. It is interesting that the majority of the RF122-unique genes were encoded by MGEs [16]. Furthermore, genes encoding well-known virulence factors such as *spa*, *clfA*, *sdrC* and *ebh* in RF122 contained premature codons and thus are pseudogenes [16]. Recently, McCarthy and Lindsay [17] confirmed that many of the *S. aureus* surface protein genes were missing or truncated in 58 strains with published sequences from various types of hosts. Thus, it is plausible that surface proteins are potential targets for horizontal gene transfer of mobile genetic elements. The Serine-Aspartate Repeat (Sdr) family is one type of the cell wall-anchored proteins. However, whether these genes are inclined to mutation like other surface protein genes has not been reported.

The Sdr proteins in *S. aureus* are members of the Microbial Surface Components Recognizing Adhesive Matrix Molecules (MSCRAMM) family encoded by the tandemly arrayed *sdrC, sdrD and sdrE* genes [18]. In addition, *sdrF, sdrG* and *sdrH* have been reported in *Staphylococcus epidermidis (S. epidermidis)* [19]. The Sdr proteins are characterized by the presence of an R region containing various numbers of the Ser-Asp dipeptides. The Sdr proteins have a similar structural organization. A signal peptide is followed by an A region which is similar in size among the different members of the Sdr family. However, they are not closely related with only 20–30% identical amino acid residues [18]. The A region is followed by B repeats. The Sdr proteins have two, three or five additional 110- to 113-residue sequences (B repeats) that are tandemly repeated in SdrC, SdrE and SdrD, respectively. The B repeats are followed by the R region. The C termini contain LPXTG motifs and hydrophobic amino acid segments. However, SdrH in *S. epidermidis* has a short 60-residue A region at its N terminus followed by the R domain without B repeats [19]. In addition, SdrH in *S. epidermidis* has a unique 277-residue C region and a C-terminal hydrophobic segment, without the LPXTG motif [19]. A few ligands for Sdr proteins in *S. aureus* have been identified: bone sialoprotein as a ligand for Bbp (bone sialo-binding protein), which is an allelic variant of SdrE [20], and beta-neurexin as a ligand for SdrC [21]. Moreover, the ligands for SdrF and SdrG in *S. epidermidis* were type I collagen and fibrinogen, respectively [22], [23].

The function of Sdr proteins in *S. aureus* remains unknown. However, there have been a few studies which reported a strong correlation between *sdr* genes of *S. aureus* and certain human diseases according to the distributional assay of *sdr* genes. Peacock et al. [24] demonstrated a strong correlation between *S. aureus* invasiveness and the presence of one of the allelic variants of the *sdrE* gene. Moreover, Trad et al. [25] reported a significantly higher prevalence of the *sdrD* gene in *S. aureus* strains responsible for bone infections. Sabat et al. [26] also showed that the *sdrD* gene was significantly associated with osteomyelitis but not with blood infections. On the other hand, there were no significant correlations of *sdrE* with blood infections and with osteomyelitis [26]. While *sdrD* was significantly associated with methicillin-resistant *S. aureus* (MRSA) strains, the *sdrE* distribution did not differ between the MSSA and MRSA strains [26]. Nevertheless, there has been little systemic research on the distribution of *sdr* genes in *S. aureus* isolates from bovine mastitis.

The traditional assay for distribution of genes involves amplifying the most conserved regions of genes, usually 100–1000 bp, in order to confirm the presence or absence of the targets [26], [27]. With the rapid development of sequencing technology and much cheaper prices for sequencing, it is now feasible to sequence whole length of a target gene or a targeted function region for identifying the presence of the gene and revealing mutations in the un-conserved regions at the same time. In the current study, therefore, the whole length of both the A region and B repeats of *sdrC, sdrD, sdrE* genes and the whole length of *sdrH* gene were amplified. Our results revealed several insertion and deletion mutation sites in these *sdr* genes. Further bio-informatics analyses showed the potential existence of horizontal gene transfer of mobile genetic elements. In addition, the correlation between the distribution of *sdr* genes in these isolates and clinical or subclinical symptoms was calculated.

## Results

### Sequence information revealed the existence of mutations in *sdr* genes in bovine mastitis isolates

To identify whether there was any difference between the specific sequences of *sdrC, sdrD, sdrE* and *sdrH* from bovine mastitis and those of the same genes in sequenced strains, sequence alignment was performed with DNAMAN software (version 6.0). As shown in Table 1, the A region and B repeats for *sdrC* in isolates from Ontario and Western Canada and the A region and B repeats for *sdrD* from all four regions shared 99.15% and 99.84% DNA sequence identity with those from the MRSA strain named TCH130, respectively. Interestingly, *sdrD* from all four regions contained a 1623_1626delATCT deletion mutation in the C-terminus of the A region, resulting in a frameshift that terminated translation at 553Leu, and loss of 832aa at the C-terminus. The A region and B repeats for *sdrE* in isolates from Ontario and Western Canada shared 98.83% DNA sequence identity with those from strain JKD6159. The full sequence of *sdrH* in isolates from Ontario and Western Canada shared 98.65% sequence identity with strains Mu3 and Mu50. The A region and B repeats for *SdrC, sdrE* and the full sequence of *sdrH* in isolates from Quebec and Eastern Canada shared 100.00%, 99.96% and 100.00% DNA sequence identity with those from strain RF122, respectively. On the other hand, the A region and B repeats for *sdrD* in isolates from Quebec and Eastern Canada shared 99.84% sequence identity with those from strain TCH130.

**Table 1:** DNA sequence identities (%) of *sdr* genes between *S. aureus* isolates in Canada and sequenced *S. aureus* strains.

Strain	Ontario & Western Canada Isolates				Quebec & Eastern Canada Isolates			
	*sdrC*(2001)*	*sdrD*(3209)	*sdrE*(2649)	*sdrH*(1233)	*sdrC*(1969)	*sdrD*(3209)	*sdrE*(2649)	*sdrH*(1299)
RF122	90.91	-	87.79	89.17	100.00	-	99.96	100.00
MRSA252	90.10	-	87.13	93.66	95.42	-	95.16	87.55
ST398	90.81	89.70	-	91.20	95.92	89.70	-	91.91
ED133	90.61	89.95	96.43	92.70	95.52	89.95	89.09	93.10
JKD6159	88.58	89.31	98.83	90.63	91.08	89.31	87.56	90.35
TCH130	99.15	99.84	96.70	99.36	90.81	99.84	86.72	89.32
MSSA476	94.99	93.68	96.32	89.84	91.12	93.68	88.56	86.05
MW2	94.94	93.62	96.32	96.80	91.07	93.62	88.56	91.18
ED98	95.53	99.60	97.63	97.71	88.94	99.60	88.19	90.80
N315, JH1, JH9, 04-02981	95.48	99.63	97.60	97.71	88.94	99.63	88.12	90.80
Mu50, Mu3	95.53	99.63	97.56	98.65	88.94	99.63	88.08	89.99
TW20	94.39	94.44	96.17	96.80	89.60	94.44	88.53	91.18
NCTC8325	94.39	94.32	-	96.72	89.60	94.32	-	91.10
COL	94.44	94.48	95.56	97.71	89.55	94.48	87.97	90.88
Newman	94.44	94.51	95.56	96.80	89.55	94.51	87.97	91.18
USA300_FRP3757USA300_TCH1516	94.39	94.44	95.63	97.16	89.50	94.44	88.04	90.36

*Nucleotide numbers of the A region and B repeats of *sdrC, sdrD* and *sdrE* and the full sequence of *sdrH* in bovine mastitis isolates.

**Figure 1:**
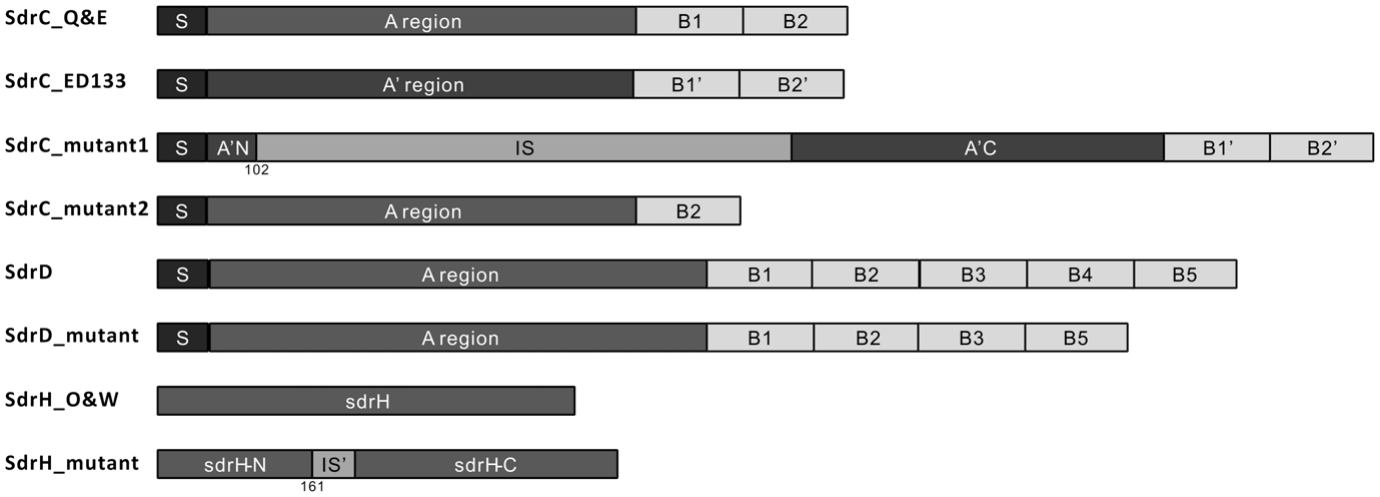
Insertion and deletion mutations in *sdr* genes of bovine mastitis-associated *S. aureus.* S represents the signal sequence while A and A' regions stand for putative ligand-binding A region. B1, B2, B3, B4, B5, B1′ and B2′ designate single B repeat. A'N and A'C are flanking regions of the insertion sequence which are identical to the N-terminus and the C-terminus of SdrC_ED133 A region, respectively; IS denotes an insertion sequence in the A region of SdrC_ED133; sdrH-N and sdrH-C represent flanking regions of the 3 repeats insertion sequence which are identical with N-terminus and the C-terminus of SdrH_O&W; IS' represents 3x repeats insertion sequence in sdrH mutant. 102 and 161 are the locations for the insertion. SdrC_Q&E represent the normal SdrC proteins in isolates from Quebec and East Canada; SdrC_mutant1 represents the insertion sequence found in SdrC in Canadian isolates E48, E49 and E50. SdrC_mutant2 represents the deletion sequence found in SdrC in Canadian isolates E5-16, CE6-14, CE16 and CE18; SdrD represents the normal SdrD proteins in Canadian isolates; SdrD_mutant represents the deletion sequence found in SdrD in Canadian isolates Q14, Q17, E7, E13, E21-24, E28-30, E45, E48, CE6-10, CE13, CE16, CE18-23, CE28, O3, O4, O18-20, CO4, CO7, CO8, CO11, CO16, CO18, CO20, O3, O4, O18-20, W9, W13, W15, W16, W20-23, W25, W32, W33, W35-37, W39, W40, CW3, CW4 and CW18; SdrH_O&W represents the normal SdrH proteins in isolates from Ontario and Western Canada; SdrH_mutant represents the insertion sequence found in SdrH in Ontario and Western Canada isolates O21-24.

Several types of insertion and deletion in specific *sdr* sequences were found (Figure 1). An insertion sequence was found in the A region of *sdrC* from three subclinical isolates of one individual cow in Eastern Canada at codon 102 (Figure 1). Interestingly, even the sequences for flanking regions of the insertion of these three isolates were different from others from the same region. The BLAST results in NCBI revealed that the flanking sequences and the “insertion sequence” in this mutant were found in the *S. aureus* strain ED133 (ST133) sequence. ED133 was isolated from ovine clinical mastitis in France [28]. Moreover, a deletion mutation was found in the B repeats of *sdrC* in 23 isolates from 14 cows in Eastern Canada (11 clinical isolates from 7 cows and 12 subclinical isolates from 7 cows), with one B repeat lost and only one B repeat remaining in the B repeats of *sdrC* (Figure 1). Similarly, one B repeat was lost in the B repeats of *sdrD* in 58 clinical and subclinical isolates from Quebec (2 subclinical isolates from 2 cows), Eastern Canada (14 clinical isolates from 12 cows and 11 subclinical isolates from 9 cows), Western Canada (3 clinical isolates from 3 cows and 16 subclinical isolates from 12 cows) and Ontario (7 clinical isolates from 7 cows and 5 subclinical isolates from 4 cows), with 4 B repeats remaining in the B repeats of *sdrD* (Figure 1). In addition, an insertion mutation was detected in the R domain of *sdrH* from 4 subclinical isolates of 2 cows in Ontario (Figure 1). The insertion sequence was 3 “DNPKPKPDPKPDP” repeats at codon 161. It seems that these 3 repeats were the product of a duplication of the same adjacent repeats in the R domain of *sdrH* mutants. However, no sequence diversity was found in *sdrE*.

### Alignment of sequencing classified bovine mastitis isolates into two types

**Figure 2:**
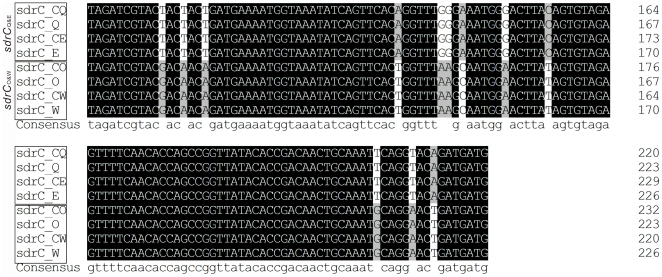
Bovine mastitis-associated *S. aureus* isolates was classified according to *sdr* genes. Alignment of a partial DNA sequence of *sdrC* gene of bovine mastitis isolates classifies *S. aureus* isolates. The same classification can be obtained by alignment of the full sequence of *sdrC* gene, as shown in Figure S1. sdrC_CQ, sdrC_CE, sdrC_CO and sdrC_CW represent *sdrC* genes from clinical isolates of Quebec, Eastern Canada, Ontario and Western Canada, respectively. sdrC_Q, sdrC_E, sdrC_O and sdrC_W represent *sdrC* genes from subclinical isolates of Quebec, Eastern Canada, Ontario and Western Canada, respectively. *sdrC_Q&E_* and *sdrC_O&W_* stand for two different types of *sdrC* gene in isolates from the Quebec and Eastern Canada region, and from the Ontario and Western Canada region, respectively.

The alignment results of *sdrC, sdrD, sdrE* and *sdrH* genes from bovine mastitis isolates indicated that the *sdrC, sdrE* and *sdrH* genes could be classified into two types. The A region and B repeats for *sdrC and sdrE* as well as *sdrH* gene in isolates from Quebec and Eastern Canada were identical, and were classified in the same type; while the A region and B repeats for *sdrC and sdrE* and the *sdrH* gene in isolates from Ontario and Western Canada shared the identical sequence but were different from isolates from Quebec and Eastern Canada. Thus, they belonged to another type. The type of *sdrC, sdrE* and *sdrH* in same isolate was identical. The *sdrC* gene was used as an example and shown in Figures 2 and S1. The *sdrC* genes from bovine mastitis isolates in Canada were classed into 2 types: One type for isolates from Quebec and Eastern Canada and another type for isolates from Ontario and Western Canada. There were no differences in amplified *sdr* PCR fragments between clinical and subclinical isolates from the same region. The A region and B repeats of *sdrD* in isolates from all four Canadian regions were identical.

### The distribution of *sdrC, sdrD, sdrE* and *sdrH* of *S. aureus* was associated with clinical and subclinical isolates of bovine mastitis

The contribution of particular binding factors to *S. aureus* pathogenesis in bovine mastitis is poorly understood. In order to find the relationship between the bovine mastitis and the distribution of *sdr* genes in *S. aureus,* the distribution of *sdrC, sdrD, sdrE* and *sdrH* of *S. aureus* between clinical and subclinical isolates of bovine mastitis was investigated. *As* shown in Table 2, the *sdrC* gene was present in all investigated isolates (n = 218). However, in 2 subclinical strains from one individual cow in Ontario (of the total 134 subclinical strains), only the *sdrC* gene (*sdrD* negative, *sdrE* negative and *sdrH* negative) was found in the *sdr* locus. Fifteen subclinical strains from 10 different cows (5 from Western Canada, 4 from Quebec and 1 from Ontario) only contained *sdrC* and *sdrH* genes (*sdrD* negative, *sdrE* negative). Almost all of the isolates contained *sdrH* gene, except for 3 subclinical isolates, with 2 from Ontario and 1 from Western Canada. All clinical isolates from Ontario and Quebec contained *sdrD* and *sdrE* genes.

**Table 2:** Distribution of *sdr* genes from clinical and subclinical isolates of bovine mastitis from different Canadian regions.

Source of isolates*	No. of isolates	***sdrC***[No.(%)]	***sdrD***[No.(%)]	***sdrE***[No.(%)]	***sdrH***[No.(%)]
CW	**18**	**18(100.0)**	**17(94.4)**	**15(83.3)**	**18(100.0)**
W	**38**	**38(100.0)**	**32(84.2)**	**20(52.6)**	**37(97.4)**
CO	**16**	**16(100.0)**	**16(100.0)**	**15(93.8)**	**16(100.0)**
O	**24**	**24(100.0)**	**19(79.2)**	**19(79.2)**	**22(91.7)**
CQ	**20**	**20(100.0)**	**17(85.0)**	**20(100.0)**	**20(100.0)**
Q	**25**	**25(100.0)**	**5(20.0)**	**19(76.0)**	**25(100.0)**
CE	**30**	**30(100.0)**	**22(73.3)**	**29(96.7)**	**30(100.0)**
E	**47**	**47(100.0)**	**43(91.5)**	**46(97.9)**	**47(100.0)**
All Clinical	**84**	**84(100.0)**	**72(85.7)**	**79(94.0)**	**84(100.0)**
All Subclinical	**134**	**134(100.0)**	**99(73.9)**	**104(77.6)**	**131(97.8)**

*CW, CO, CQ and CE represent clinical isolates from Western Canada, Ontario, Quebec and Eastern Canada, respectively. W, O, Q and E represent subclinical isolates from Western Canada, Ontario, Quebec and Eastern Canada, respectively.

A significant association between the *sdrC*-positive, *sdrH*-positive, *sdrD*-negative, *sdrE*-negative gene profile and subclinical strains was found (15/119 versus 0/84; Fisher's exact test; P = 0.0006). Among the tested isolates, the presence of *sdrD* was significantly associated with clinical isolates (99/35 versus 72/12; Fisher's exact test; P = 0.0431), especially in isolates from Quebec (5/20 versus 17/3; Fisher's exact test; P<0.0001). In addition, the presence of *sdrE* was significantly associated with clinical isolates (104/30 versus 79/5; Fisher's exact test; P = 0.0011), especially in isolates from Quebec (19/6 versus 20/0; Fisher's exact test; P = 0.0265) and Western Canada (20/18 versus 15/3; Fisher's exact test; P = 0.0386).

### Gene phylogenetic trees, based on *sdrC, sdrD, sdrE* and *sdrH*, revealed evolutional relationships among *S. aureus* strains and mastitis isolates

In order to better understand the relationship between the variation of *sdrC, sdrD, sdrE* and *sdrH* and the evolution of *S. aureus* strains, gene phylogenetic trees of *sdrC, sdrD, sdrE* and *sdrH* were constructed, using both the A region and B repeats of *sdrC, sdrD, sdrE* and full sequence of *sdrH* of 21 sequenced *S. aureus* strains and 218 mastitis isolates.

**Figure 3:**
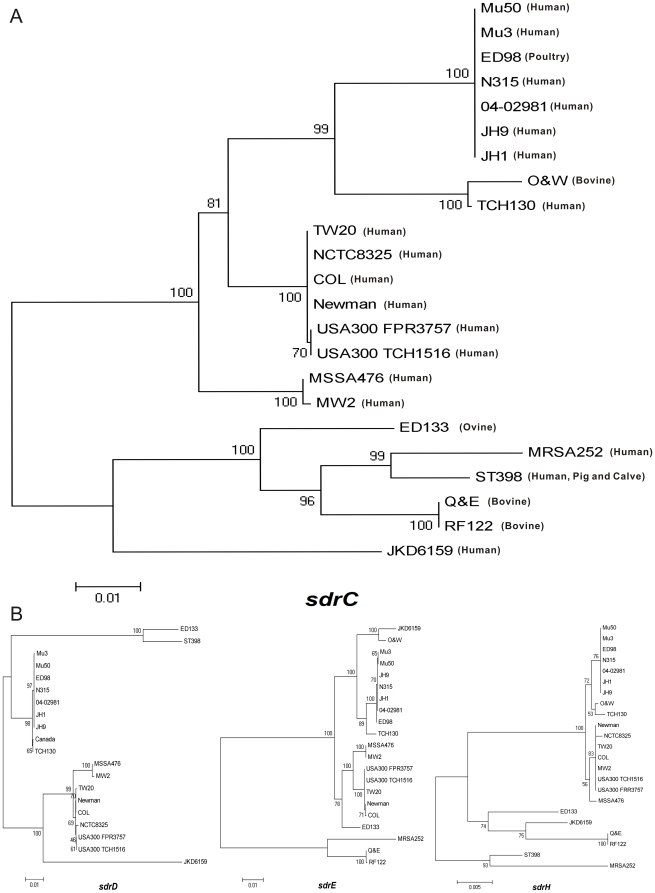
Phylogenetic trees of *sdr* genes revealed the evolutional relationship among *S. aureus strains* and isolates. The graph was constructed by using MEGA software (version 4.0) with the NJ method and bootstrap values were provided as percents over 1000 replications. Alignment gaps were considered complete deletion. A) Phylogenetic tree of *sdrC* gene. Q&E represents the isolates from Quebec and Eastern Canada; O&W represents the isolates from Ontario and Western Canada. B) Phylogenetic trees of *sdrD, sdrE* and *sdrH* genes. Canada in the *sdrD* phylogenetic tree denotes the isolates from all four Canadian regions.

As shown in Figure 3A, the phylogenetic tree of *sdrC* divided strains and isolates into several clusters. One main cluster included strains Mu50, ED98, Mu3, N315, 04-02981, JH1, JH9, TCH130 and Ontario and Western Canada isolates. Another main cluster included strains Newman, COL, NCBC8325, TW20, USA300 TCH1516 and USA300 FPR3757 and this cluster was more conserved than the former cluster. Ovine-associated strain ED133 [28], bovine-associated strain RF122 [16], swine-associated strain ST398 [29], human-associated strain MRSA252 [30] and Quebec and Eastern Canada isolates were highly divergent from the above two clusters but showed phylogenetical similarity to one another. Strain JKD6159 [31] had the highest divergence from all other strains and isolates.

The phylogenetic tree of *sdrD* showed similar clustering to that of *sdrC* (Figure 3B), but one main cluster, which including strains Mu50, ED98, Mu3, N315, 04-02981, JH1, JH9, TCH130 and Ontario and Western Canada isolates, was more conserved than another main cluster, which including strains Newman, COL, NCBC8325, TW20, USA300 TCH1516 and USA300 FPR3757. Strains ED133 and ST398 were similar with each other and JKD6159 was still the most genetically diverse *S. aureus* strain. Strains RF122 and MRSA252 did not have *sdrD* and were not included for constructing the phylogenetic tree. The *sdrE* and *sdrH* phylogenetic trees were also similar in organization to those of *sdrC* and *sdrD* (Figure 3B), however, strain JKD6159 appeared to be closely related to Ontario and Western Canada isolates. The *sdrE* gene seems less variable than *sdrC* and *sdrD* genes according to the *sdrE* phylogenetic tree. Strains ST398 and NCTC8325 were not included for constructing the phylogenetic tree of *sdrE*, due to their lack of *sdrE* sequences.

The concatenated sequences of the seven multilocus sequence typing (MLST) of all the 21 *S. aureus* strains used in this study were also used for constructing the phylogenetic tree of strains, as described [30]. The results from the *sdr* phylogenetic trees (Figure 3) were in general agreement with the one from MLST typing (Figure S2). Figure 3 showed the relationship among strains MRSA252, ED133, ST398, RF122 and JKD6159. However, such relationship was not evident in the MLST typing.

## Discussion

One of the novel findings from this study was the discovery of possible horizontal gene transfer between bovine *S. aureus* isolates and strains from other species. The A region and B repeats of *sdrC, sdrE* and the full sequence of *sdrH* in bovine mastitis isolates from Quebec and Eastern Canada shared 100.00%, 99.96% and 100.00% sequence identities with those in RF122, respectively (Table 1). However, the A region and B repeats of *sdrD* from all Canadian regions shared 99.84% sequence identities with genes from TCH130. RF122 is a bovine mastitis strain, while TCH130 is a human-associated strain (Table 3). Some bovine mastitis isolates from Quebec and Eastern Canada did not contain *sdrD* gene, while others did. These results suggested isolates from Quebec and Eastern Canada without *sdrD* might be homologous to RF122 strain, since RF122 does not contain the *sdrD* gene. The *sdrD* in *S. aureus* isolates from Quebec and Eastern Canada could be obtained through the horizontal transfer from TCH130 or a strain related to TCH130, since these two *sdrD* were almost identical. Alternatively, a genetic drift might have occurred in isolates with from Quebec and Eastern Canada and lost *sdrD* gene. However, only a gene with minor or no function may be lost due to a genetic drift [32]. Considering the fact that the *sdrD* gene is strongly associated with serious human diseases [25], [26], [27] and the clinical mastitis as shown in this study, it is unlikely that the genetic drift is responsible for our observed sequence diversity of *sdrD*. The A region and B repeats for *sdrC* and *sdrE* in isolates from Quebec and Eastern Canada also showed 95.42% and 95.16% of sequence identities with those of MRSA252, respectively (Table 1). RF122 and MRSA252 have been reported to share 14 different DNA sequence blocks [33]. In addition, RF122 and MRSA252 were the only 2 published strains which do not contain a *sdrD* gene, suggesting that these two strains were evolutionarily similar. Interestingly, *sdrC* from Quebec and Eastern Canada isolates contained premature stop codons and thus was a pseudogene, as was also reported for RF122 [16]. On the other hand, the A region and B repeats for *sdrC* in Quebec and Eastern Canada isolates also showed 95.52% and 95.92% sequence identities with those of strains ED133 (an ovine mastitis strain) and ST398 (a swine strain), respectively (Table 1 and Table 3). The *sdrC* gene in strains ED133 and ST398 was not truncated. Therefore, the *sdrC* locus in bovine strains and isolates seems not essential for inducing bovine mastitis.

**Table 3:** *S. aureus* strains evaluated in this study.

Strain	MLST Type*	Geographic origin	Year	Comments**	Host and diseases
Mu50	ST5	Japan	1997	HA-MRSA/VISA	Human with Vancomycin resistance
Mu3	ST5	Japan	1996	MRSA/hetero-VISA	Human with Pneumonia
ED98	ST5	Northern Ireland	1996–1997	N/A***	Poultry with BCO
N315	ST5	Japan	1982	HA-MRSA/VSSA	Human
04–02981	ST225	Köln, Germany	2004	MRSA	Human
JH1	ST105	New York, USA	2000	MRSA/VSSA	Human, the earliest isolate of JH9
JH9	ST105	New York, USA	2000	MRSA/VISA	Human with Vancomycin resistance
TCH130	ST72	Houston, USA	2001	MRSA	Human with pneumonia
TW20	ST239	London, UK	2003	MRSA	Human with Bacteremia
NCTC8325	ST8	Colindale, UK	1940s	MSSA	Human with Sepsis
COL	ST250	Colindale, UK	1961	MRSA	Human with Penicillinase-negative
Newman	ST8	UK	1952	MSSA	Human with tubercular Osteomyelitis
USA300-FPR3757	ST8	San Francisco, USA	2002–2004	CA-MRSA	Human with HIV-positive
USA300-TCH1516	ST8	San Francisco, USA	2002–2004	CA-MSSA	Human with sepsis
MSSA476	ST1	Oxford, UK	1998	CA-MSSA	Human with Osteomyelitis and Bacteremia
MW2	ST1	North Dakota, USA	1998	CA-MRSA	Human with septic arthritis and septicaemia
ED133	ST133	France	1997	N/A	Ovine mastitis
MRSA252	ST36	Oxford, UK	1997	HA-MRSA	Human with Septicemia
ST398	ST398	Netherlands	2006	MRSA	Human with Endocarditis, Also highly infect pigs and calves.
RF122	ST151	Ireland	1993	MSSA	Bovine mastitis
JKD6159	ST93	Australia	2003	CA-MRSA	Human with Septicemia
CW1-18	N/A	Canada	2007–2008	MSSA	Bovine mastitis
W1-38	N/A	Canada	2006–2007	MSSA	Bovine mastitis
CO1-16	N/A	Canada	2007	MSSA	Bovine mastitis
O1-24	N/A	Canada	2007	MSSA	Bovine mastitis
CQ1-20	N/A	Canada	2007	MSSA	Bovine mastitis
Q1-25	N/A	Canada	2007	MSSA	Bovine mastitis
CE1-30	N/A	Canada	2007	MSSA	Bovine mastitis
E1-47	N/A	Canada	2007	MSSA	Bovine mastitis

*MLST represents multilocus sequence typing.

**HA-MRSA means hospital-acquired methicillin-resistant *S. aureus*; CA-MSSA is the community-acquired methicillin-sensitive *S. aureus*; VISA is vancomycin-intermediate level-resistant *S. aureus*; VSSA represents vancomycin sensitive *S. aureus*.

***N/A, Not available.

Possible horizontal gene transfer between bovine *S. aureus* isolates and strains from other species was also evident in Ontario and Western Canada isolates. The *sdrC* and *sdrD* genes in isolates from Ontario and Western Canada shared 99.15% and 99.84% (Table 1) of sequence identities with those from *S. aureus* strain TCH130, which came from a 2 year old child with pneumonia (Human Microbiome Project, http://www.ncbi.nlm.nih.gov/nuccore/ACHD00000000). Some isolates from Ontario and Western Canada contained both *sdrC* and *sdrD,* while others only contained *sdrC,* not *sdrD.* The *sdrE* gene in isolates from Ontario and Western Canada shared 96.70% and 98.83% of sequence identities with those from TCH130 and JKD6159, respectively (Table 1). The *sdrC, sdrD, sdrE* and *sdrH* gene in isolates from Ontario and Western Canada also shared an average of 95.51%, 99.62%, 97.60% and 98.02% sequence identify with genes from the second cluster which including strains ED98, Mu3, Mu50, N315, JH1, JH9 and 04-02981, respectively. *S. aureus* ED98 was isolated from a chicken with bacterial chondronecrosis with ostemyelitis (BCO) in Ireland [7], [8] and the others were human-associated *S. aureus* strains (Table 3). The identical *sdrC, sdrD, sdrE,* and *sdrH* genes between the human-associated *S. aureus* strains and ED98 suggest that HGT may have occurred among them.

Insertion and deletion were detected in the A region and B repeats of *sdrC* and *sdrD* genes (Figure 1). Some of them may be due to mobile genetic elements. An insertion sequence in the *sdrC* A region was found to be similar to the C-terminal region of *Enterococcus faecium* transposase IS1216V (EMBL: L40841), sharing 46% identity. In addition, its organizational structure was similar to transposon IS1272 [34]. As shown in Figure 1, the *sdrC* in this mutant from an Eastern Canada cow was totally different from that of other cows in the same region. Interestingly, the flanking sequences of this insertion within *sdrC* and the “insertion sequence” in this mutant were found in the *S. aureus* strain ED133 (ST133) sequence [28]. However, the insertion sequence in ED133 genome was outside of the A region and was called “putative insertion element protein”. It was plausible that the putative insertion element helped to move the *sdrC* of ED133 strain into a bovine mastitis isolate. If this should be the case, our report is the first to show the horizontal gene transfer from an ovine mastitis *S. aureus* strain to a bovine mastitis isolate. Horizontal gene transfer had been reported before among *S. aureus* strains from different hosts, for example, between strains of human and poultry [7], or between strains of human and bovine [33].

Another insertion was found in the R domain of *sdrH* in *S. aureus* isolates. The insertion sequence in *sdrH* mutants was three conserved “DNPKPKPDPKPDP” repeats at codon 161. *sdrH* gene was first reported in *S. epidermidis* [19]. By analyzing the putative *sdrH* genes of genomes of 21 sequenced *S. aureus* strains, it was found that the structure of *sdrH* gene in *S. aureus* was similar to that of in *S. epidermidis*. The insertion mutation seems a product of duplication of the 3 conserved “DNPKPKPDPKPDP” repeats as a whole unit in the wild type sdrH gene.

One deletion was detected in *sdrC* and *sdrD* genes of *S. aureus* isolates, with one B repeat lost. Bacterial simple sequence repeats (SSRs) are prone to high rates of mutation through slipped strand mispairing that result in expansions or contractions in the number of repeat units [35], [36]. Hence, the loss of B repeat could be due to the slip-stand mispairing. One study of the B repeats of SdrF, a *S. epidermidis* surface protein containing four B repeats, showed that a single B repeat of *S. epidermidis* 9491 retained the capacity to bind to its ligand [22].

The phylogenetic trees of *sdrC, sdrD, sdrE* and *sdrH* consistently showed the relationship between our *S. aureus* isolates and the published *S. aureus* strains. As shown in Figure 3, the 21 published *S. aureus* strains and our isolates were divided into several clusters by *sdr* genotyping. In the *sdrC* phylogenetic tree (Figure 3A), the *S. aureus* isolates from Quebec and Eastern Canada were phylogenetically similar to strain TCH130, while the Ontario and Western Canada isolates were homologous with strain RF122, suggesting that the *S. aureus* isolates in Canada had two totally different ancestors. In addition, MRSA252 and three other strains (ST398, RF122 and ED133) seem to share a common ancestor. This notion was also supported by the results of *sdrD, sdrE* and *sdrH* phylogenetic trees. Strain JKD6159 showed the highest divergence from other strains or isolates in *sdrC, sdrD* and *sdrE* phylogenetic trees, suggesting that JKD6159 was in the farthest evolutional end among the 21 strains. This was consistent with the fact that JKD6159 was a distant strain from Australian [31] (Table 3). A previous study showed that the genome from strain 04-02981 was co-linear with N315 and JH1 [37], which were confirmed in this study. The results from the phylogenetic trees indicated that the first main cluster containing strains Mu50, ED98, Mu3, N315, 04-02981, JH1, JH9, TCH130 and Ontario and Western Canada isolates was less conservative in *sdrC,* but more conservative in *sdrD* and equally conservative in *sdrE* and *sdrH,* in comparison with another main cluster containing strains Newman, COL, NCBC8325, TW20, USA300 TCH1516 and USA300 FPR3757. Our results suggested that these two main clusters have developed different strategies for the *sdr* gene evolution. The function of Sdr proteins in *S. aureus* pathogenesis remains unknown. Thus, it is not feasible at this time to postulate how the sequence diversity of *sdr* genes affects biological functions of the translated proteins. In addition, an evolutionary relationship should exist among *S. aureus* strains from different species. However, such a relationship was less clear in the phylogenetic tree using MLST typing in comparison with *sdr* phylogenetic trees.

Another major finding from this study was a significant association between the presence of *sdrE* and clinical strains (104/30 versus 79/5; Fisher's exact test; P = 0.0011). Another significant association was also found between the *sdrC*-positive, *sdrH*-positive, *sdrD*-negative, *sdrE*-negative gene profile and subclinical isolates (15/119 versus 0/84; Fisher's exact test; P = 0.0006), suggesting that these isolates had a substantially decreased potential to establish clinical bovine mastitis. Our result was supported by Sabat et al. [26]. They showed that the *sdrC*-positive, *sdrD*-negative, *sdrE*-negative gene profile was not found in the strains collected from bone infections [26]. A third significant association was found between the *sdrD* positive gene profile and clinical isolates, suggesting that *sdrD* was important for the pathogenesis of bacteria but not essential for bacterial survival, since some isolates did not have *sdrD*. A previous study [26] showed that there was a strong association between the presence of the *sdrD* gene and MRSA responsible for bone infections. In addition, Trad et al. [25] showed a significantly higher prevalence of *sdrD* in bone infection isolates than in nasal isolates. South African methicillin-susceptible *S. aureus* (MSSA) isolates were more likely than MRSA isolates to carry virulence gene *sdrD* [27]. The same study also showed that MRSA isolates were more likely than MSSA isolates to carry genes for *sdrC, sdrD* and *sdrE* [27]. This study was the first time to show the distribution of *sdrH* gene in *S. aureus*. Although our initial BLAST results indicated the existence of *sdrH* in *S. aureus,* this report confirmed the presence of *sdrH* genes in the bacteria.

In summary, *S. aureus* strains from bovine mastitis (RF122 and our isolates), ovine mastitis (ED133), pig, calves and human infections (ST398), poultry with BCO (ED98) and human MRSA (TCH130, MRSA252, Mu3, Mu50, N315, 04-02981, JH1 and JH9) were highly associated with one another. It is proposed that horizontal gene transfer might be responsible for this association. The presence of insertion mutation and deletion mutation in the *sdr* genes suggested that the *sdr* genes were variable. These findings are crucial for better understanding the emergence of traits such as increased virulence or antibiotic resistance, together with the forces driving pathogen spread.

## Materials and Methods

### Bacterial strains and isolates

Eighty four clinical isolates and 134 subclinical bovine mastitis-associated *S. aureus* isolates from 149 cows were used in this study and different isolates from a same cow were isolated from different sampling points. They were from 4 different regions in Canada, including Western Canada (18 clinical and 38 subclinical isolates), Ontario (16 clinical and 24 subclinical isolates), Quebec (20 clinical and 25 subclinical isolates) and Eastern Canada (30 clinical and 47 subclinical isolates) (Table 2). These were obtained from the Canadian Bovine Mastitis Research Network (CBMRN) and all isolates are MSSA [38]. In addition, published sequences of 21 strains were used as reference strains for comparison analyses. These strains included Mu3 (AP009324), Mu50 (BA000017), TW20 (FN433596), N315 (BA000018), NCTC8325 (CP000253), ED98 (CP001781), COL (CP000046), JKS6159 (CP002114), MRSA252 (BX571856), MSSA476 (BX571857), ED133 (CP001996), MW2 (BA000033), RF122 (AJ938182), USA300 FPR3757 (CP000255), USA300 TCH1516 (CP000730), JH1 (CP000736), JH9 (CP000703), Newman (AP009351), 04-02981 (CP001844), TCH130 (NZ ACHD00000000) and ST398 (AM990992).

### Genomic DNA extraction

*S. aureus* isolates were grown on 3 ml nutrient broth medium overnight (37°C) before being harvested by centrifugation at 10,000 g for 1 min. The pellet was resuspended in 300 ul of the MicroBead solution containing 20 ul of lysostaphin (1 mg/ml; Sigma Aldrich, St. Louis, MO). The genomic DNA was extracted using an UltraClean® Microbial DNA Isolation Kit (Mo Bio Laboratories, Carlsbad, CA) according to manufacturer's instructions. DNA concentration was determined by an ND-1000 spectrophotometer (NanoDrop Technologies, Wilmington, DE). The DNA samples were stored at −20°C until being used for subsequent analyses.

### Comparative genome analysis, primer design and PCR amplification

The genomes of 21 strains of *S. aureus* from different hosts were available in the NCBI nucleotide data base. All of them contained *sdrC* and *sdrH,* and nearly all of them contained *sdrD* and *sdrE* (strain NCTC8325 and ST398 did not contain *sdrE*, while strains MRSA252 and RF122 did not have *sdrD*). Alignment of the *sdrC, sdrD, sdrE* and *sdrH* sequence of 21 *S. aureus* strains showed that both C terminal and N terminal regions in both A region and B repeats of *sdrC, sdrD, sdrE* and whole *sdrH* gene were conserved. Consequently, primers were designed for these regions. PCR amplifications were performed in a Mastercycler (Eppendorf AG, Hamburg, Germany) with Crimson *Taq* DNA polymerase (New England Biolabs Inc. Pickering, ON.). The reaction tubes contained 20 ng of genomic DNA, 0.5 uM of each forward and reverse primer, 2.5 mM MgCl_2_, 0.2 mM deoxynucleoside triphosphates (dNTPs), 2 U of Crimson *Taq* DNA polymerase (New England Biolabs Inc) and 5 ul *Taq* buffer in a total volume of 25 ul. Conditions for the each reaction were as follows: 95°C for 5 min; 32 cycles of 95°C for 30 sec, different annealing temperatures (Table 1) for 30 sec, and 72°C for 200 sec; 72°C for 7 min; and final hold at 4°C. PCR products were analyzed by 1% agarose gel electrophoresis.

### Sequence alignment among *sdrC, sdrD, sdrE* and *sdrH* genes of bovine mastitis-associated *S. aureus* produced by PCR

The PCR products for *sdrC, sdrD, sdrE* and *sdrH* genes were all submitted to the McGill University and Génome Québec Innovation Centre for sequencing. The Centre offers a Sanger Sequencing Service using Applied Biosystem's 3730xl DNA Analyzer technology. The A region and B repeats of *sdrC*, *sdrD* and *sdrE* genes were amplified separately with different primer pairs (Table 4). All of the mutants found in this research were amplified and sequenced at least twice in order to rule out the possibility of sequence errors. The DNA sequences of different *sdr* genes were aligned with the DNAMAN software (version 6.0), using the multiple sequence alignment program, to find differences among the sequenced isolates. The full alignment type of the optimal alignment method was used with default parameters. The DNAMAN uses ClustalW algorithm for optimal alignment. Our preliminary result indicated the existence of *sdrH* in *S. aureus* isolates, as shown in published sequences in NCBI. Therefore, *sdrH* was also analyzed in our study.

**Table 4:** Primers used in PCR amplification of DNA sequence.

PCR product(size)	Primer	Primers Sequence	Annealing Temperatures
***sdrC* A region (1356 bp)**	*sdrC* -A-F	5′-GTGGTCATGAAGCTAAAGCGG-3′	56°C
	*sdrC* -A-R	5′-TCTTTTGGTCGCCATTAGCAG-3′	
***sdrD* A region (1569 bp)**	*sdrD* -A-F	5′-GGAACCAAGAAGCAAAGGCTG-3′	56°C
	*sdrD* -A-R	5′-CTTCTTGACCAGCTCCGCCAC-3′	
***sdrE* A region (1663 bp)**	*sdrE* -A-F	5′-GGAACCAAGAAGCTAAAGCTG-3′	56°C
	*sdrE* -A-R	5′-ACTTTTCTTCAGGTTTAACAG-3′	
***sdrC* B repeats (691 bp)**	*sdrC* -B-F	5′-CTGCTAATGGCGACCAAAAGA-3′	44°C
	*sdrCDE*-R	5′-TCTGATGTTTCTTCTTC-3′	
***sdrD* B repeats (1690 bp)**	*sdrD* -B-F	5′-GTGGCGGAGCTGGTCAAGAAG-3′	44°C
	*sdrCDE*-R	5′-TCTGATGTTTCTTCTTC-3′	
***sdrE* B repeats (1027 bp)**	*sdrE* -B-F	5′-CTGTTAAACCTGAAGAAAAGT-3′	44°C
	*sdrCDE*-R	5′-TCTGATGTTTCTTCTTC-3′	
***sdrH* full sequence (1272 bp)**	*sdrH* -F	5′-ATGTCATATCATTGGTTTAAG-3′	56°C
	*sdrH* -R	5′-TTATCGTCGCTGTGATTCGTT-3′	

### Construction of gene phylogenetic trees

In order to identify the relationships of *sdr* genes in *S. aureus* from different hosts, a phylogenetic approach was used. The DNA sequence of the A region and B repeats of *sdrC, sdrD*, *sdrE* genes and the full sequence of *sdrH* gene from bovine mastitis isolates as well as the genome sequences of 21 *S. aureus* strains were used to construct their phylogenetic trees. A phylogenetic tree was also constructed for the 21 sequenced *S. aureus* strains, using the seven MLST loci. The DNA sequences were aligned using the ClastalW2 program with default parameters followed by manual inspection. Phylogenetic trees were constructed with the neighbor-joining (NJ) method and bootstrap values were provided as percents over 1000 replications, utilizing the Molecular Evolutionary Genetics Analysis (MEGA) software version 4.0 on default setting [39]. Alignment gaps were considered complete deletion.

## Supporting Information

Figure S1 **Bovine mastitis-associated** ***S. aureus*** **isolates was classified according to** ***sdr*** **genes.** Alignment of the full DNA sequence of *sdrC* gene of bovine mastitis isolates classifies *S. aureus* isolates**.** sdrC_CQ, sdrC_CE, sdrC_CO and sdrC_CW represent *sdrC* genes from clinical isolates of Quebec, Eastern Canada, Ontario and Western Canada, respectively. sdrC_Q, sdrC_E, sdrC_O and sdrC_W represent *sdrC* genes from subclinical isolates of Quebec, Eastern Canada, Ontario and Western Canada, respectively.
(TIF)

Figure S2 **Phylogenetic organization of 21 sequenced** ***S. aureus*** **strains was demonstrated using the seven MLST loci.** The graph was constructed by using MEGA software (version 4.0) with the NJ method and bootstrap values were provided as percents over 1000 replications. Alignment gaps were treated with the complete deletion option.
(TIF)
