# Supplementary figures and images for: Sequence Diversities of Serine-Aspartate Repeat Genes among Staphylococcus aureus Isolates from Different Hosts Presumably by Horizontal Gene Transfer
Source: PLoS One. 2011 May 20;6(5):e20332. doi: 10.1371/journal.pone.0020332 (PMC3098876; doi:10.1371/journal.pone.0020332)

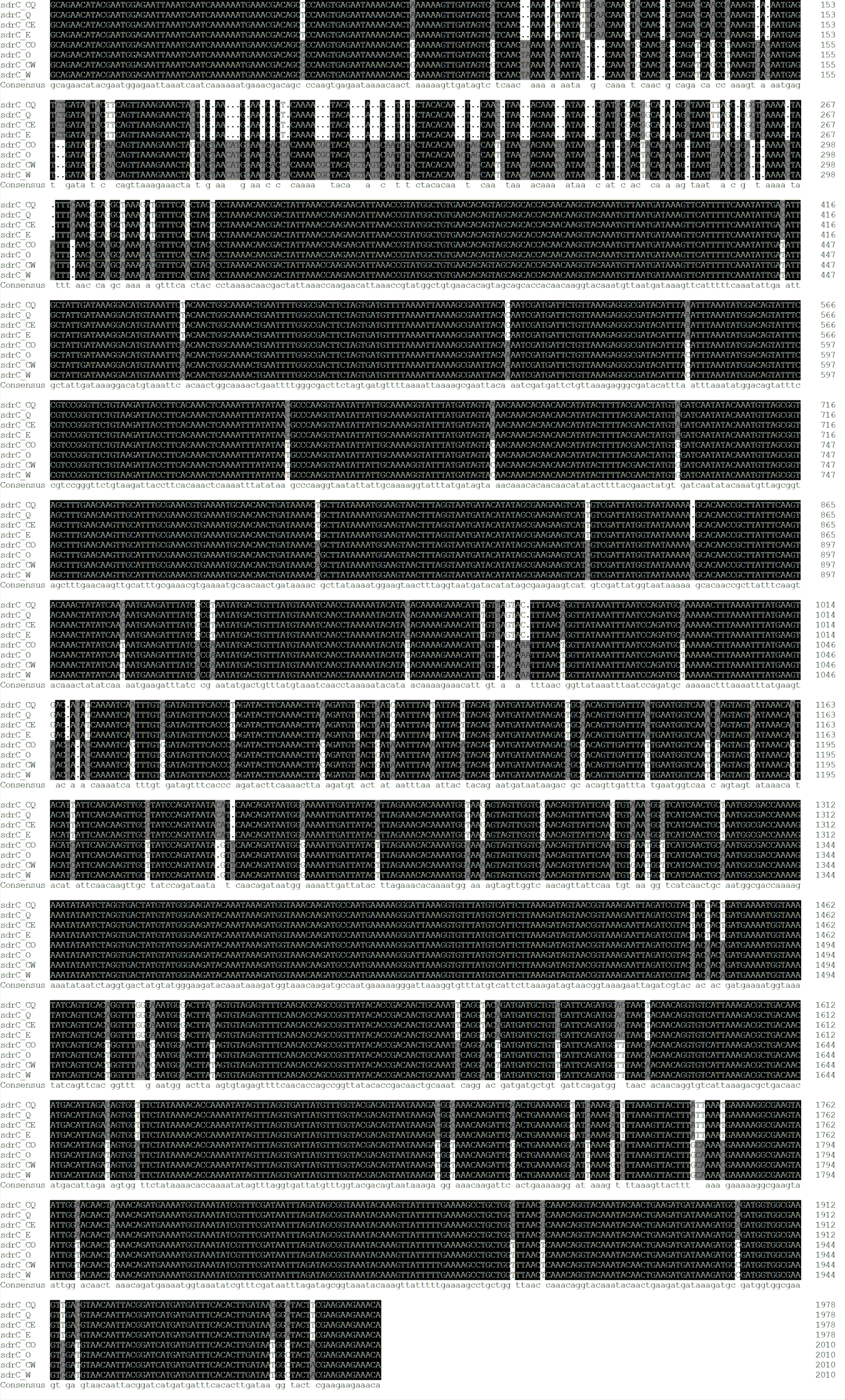

Supplement: Figure S1 — Bovine mastitis-associated S. aureus isolates was classified according to sdr genes. Alignment of the full DNA sequence of sdrC gene of bovine mastitis isolates classifies S. aureus isolates. sdrC_CQ, sdrC_CE, sdrC_CO and sdrC_CW represent sdrC genes from clinical isolates of Quebec, Eastern Canada, Ontario and Western Canada, respectively. sdrC_Q, sdrC_E, sdrC_O and sdrC_W represent sdrC genes from subclinical isolates of Quebec, Eastern Canada, Ontario and Western Canada, respectively. (TIF) [file pone.0020332.s001.tif]
